# Aberrant intra‐epithelial lymphocytes cause enterocyte cell death in refractory celiac disease by CD103‐β7‐receptor‐mediated granzyme‐B degranulation which can be restored by etrolizumab

**DOI:** 10.1002/cti2.70099

**Published:** 2026-05-14

**Authors:** Daan A R Castelijn, Jolanda M W van de Water, Laura R de Baaij, Bridget S McGlinchy, Michelle van Leeuwen, Sander P J Joosten, Nikita Alberts, Margherita Rossi, Martine Reijm, Jolien C Hollander, Petra Bonnet, Yvonne M C Kooy‐Winkelaar, Frits Koning, Steven T Pals, Gerd Bouma, Chris J L M Meijer, Hetty J Bontkes, Chris J J Mulder, Saskia A G M Cillessen

**Affiliations:** ^1^ Department of Gastroenterology and Hepatology, Amsterdam Gastroenterology Endocrinology and Metabolism Research Institute Amsterdam University Medical Centers Amsterdam The Netherlands; ^2^ Department of Pathology, Amsterdam Gastroenterology Endocrinology and Metabolism Research Institute Amsterdam University Medical Centers Amsterdam The Netherlands; ^3^ Department of Internal Medicine St. Antonius Hospital Nieuwegein The Netherlands; ^4^ Department of Clinical Chemistry, Amsterdam Institute for Infection and Immunity Amsterdam University Medical Centers Amsterdam The Netherlands; ^5^ Department of Immunohematology and Blood Transfusion Leiden University Medical Center Leiden The Netherlands

**Keywords:** autoimmunity, cell death, enteropathy, intestinal inflammation

## Abstract

**Objectives:**

Refractory celiac disease type II (RCDII) is an intestinal tumor of aberrant intra‐epithelial T‐lymphocytes (IEL). The severe enteropathy found in RCDII is caused by aberrant IEL that exert cytotoxicity against enterocytes. In this study, we investigated the cell death mechanism responsible for villous atrophy in RCDII.

**Methods:**

Aberrant IEL were isolated from duodenal biopsies of RCDII patients. Enterocyte and RCDII patient‐derived cell lines and human small intestinal organoids were used. mRNA expression was determined with reverse transcriptase‐multiplex ligation‐dependent probe amplification. Protein expression, degranulation and enterocyte killing were measured using flow cytometry, immunofluorescence or bright field microscopy. Secretion of granzyme‐B was detected by enzyme immunoassay.

**Results:**

Levels of granzyme‐B expression were significantly upregulated in aberrant IEL of RCDII patients compared to patients with celiac disease (CD) on gluten‐free diet (*P* = 0.0001) and correlated with severity of villous atrophy and clinical response to therapy. Killing of intestinal epithelial cells was caused by granzyme‐B. For granzyme‐B degranulation and subsequent cytotoxicity, cell–cell binding via the CD103‐receptor, which was upregulated on aberrant IEL, was essential. In a preclinical model, aberrant IEL migrated to the intestinal organoids and induced organoid disintegration and CD103‐dependent cell death. Targeting CD103‐heterodimeric partner β7 with therapeutic monoclonal antibody etrolizumab prevented enterocyte cell killing and resulted in survival of organoids.

**Conclusion:**

Killing of enterocytes in RCDII patients depends on degranulation of granzyme‐B by aberrant IEL through CD103‐β7 binding. By blocking this interaction, etrolizumab restores the intestinal epithelium and therefore should be considered as potential therapy for RCDII patients.

## Graphical abstract

Targeting the cell death mechanism responsible for villous atrophy in RCDII. Aberrant IEL cause mucosal damage in RCDII by granzyme‐B‐induced apoptosis of enterocytes through CD103‐β7 binding. Treatment with anti‐β7 monoclonal antibody etrolizumab inhibits granzyme‐B‐induced enterocyte killing and restores viability of the intestinal mucosa.

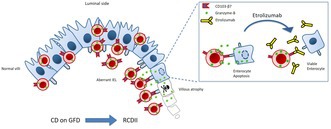

## Introduction

Refractory celiac disease type II (RCDII) is characterised by a severe enteropathy in the presence of an expansion of aberrant intra‐epithelial T‐lymphocytes (IEL). Similar to active celiac disease (CD), enterocyte cell death is a key feature of RCDII ultimately leading to symptoms of malabsorption, wasting and diarrhoea.^1^ The extent of intestinal damage is represented by the Marsh classification, ranging from intra‐epithelial lymphocytosis (Marsh 1) to crypt hyperplasia (Marsh 2) and villous atrophy (Marsh 3).^2^ The villous atrophy found in active CD is caused by an autoimmune reaction to gluten ingestion. Gluten‐free diet (GFD) is the only available treatment for patients with CD and usually results in mucosal recovery.^3^ However, in a small fraction of CD patients, RCDII originates if the severe enteropathy persists or recurs despite strict adherence to GFD.^4^ Patients with RCDII are currently treated with cladribine (2‐chlorodeoxyadenosine) and/or autologous stem cell transplantation, but only a subset of the patients responds to these therapies showing restoration of villous structure.^5^, ^6^ Moreover, RCDII can transform into a highly aggressive enteropathy‐associated T‐cell lymphoma (EATL). As a consequence, patients with RCDII have a poor prognosis, reflected in a 5‐year survival rate of approximately 50%.^7^, ^8^

In both treated and active CD, total IEL numbers are significantly increased, but in RCDII specifically the percentage of aberrant IEL is elevated (> 20%). These lineage‐negative cells express intracellular CD3 and surface CD103(αE), but lack cell‐surface expression of the T‐cell receptor (TCR)‐CD3 complex, CD4 and CD8.^9^ In addition, aberrant IEL generally display monoclonal TCR‐γ gene rearrangement, indicating their clonal expansion.^10^ Accumulation of these abnormal cells in the gut is caused by interleukin‐15 (IL‐15) regulated overexpression of the anti‐apoptotic proteins Bcl‐2 and Bcl‐X_L_.^11^ The aberrant IEL can induce massive enterocyte killing leading to villous atrophy in the small intestine.^12^ However, the mechanism responsible for enterocyte cell death is poorly understood.

In patients with active CD, apoptosis of enterocytes by CD8^+^ T‐lymphocytes is caused by granzyme‐B.^13^ Granzyme‐B is a serine protease which is predominantly released by CD8^+^ T cells and NK cells to eliminate target cells.^14^ Previously, it was shown that granzyme‐B transcription was increased in duodenal biopsies of patients with RCDII.^15^ In addition, higher serum levels of soluble granzyme‐B were observed in RCDII patients in comparison with patients with active CD.^16^ The aim of this study was therefore to explore the involvement of granzyme‐B in the killing of enterocytes by aberrant IEL in duodenal biopsies of RCDII patients in order to identify the cell death mechanism responsible for the development of severe villous atrophy.

## Results

### Granzyme‐B expression is upregulated in aberrant IEL of patients with RCDII and is positively correlated with severity of villous atrophy

**Figure 1:**
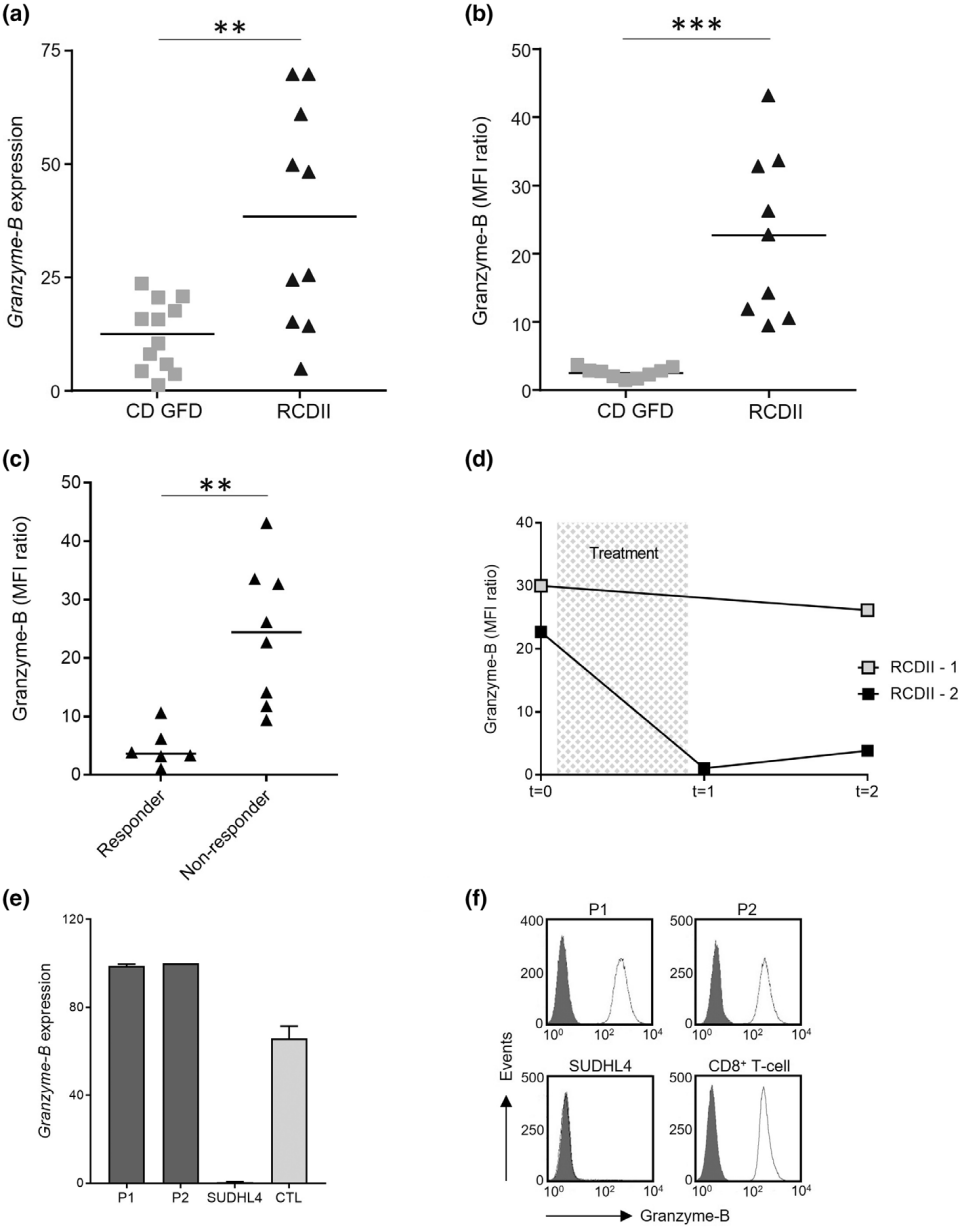
Granzyme‐B expression is upregulated in aberrant intra‐epithelial T‐lymphocyte (IEL) of refractory celiac disease type II (RCDII) patients and is positively correlated with severity of villous atrophy. **(a)** *Granzyme‐B* expression in aberrant IEL of RCDII patients and celiac disease (CD) patients on gluten‐free diet (GFD), measured by reverse transcriptase‐multiplex ligation‐dependent probe amplification (RT‐MLPA). **(b)** Protein expression of granzyme‐B in aberrant IEL of RCDII patients and patients with CD on GFD. **(c)** Granzyme‐B protein expression in aberrant IEL of non‐responder RCDII patients with persistent villous atrophy and responder RCDII patients with mucosal recovery after treatment. **(d)** Follow‐up of granzyme‐B protein expression in aberrant IEL of one responder and one non‐responder RCDII patient after treatment with cladribine (*t* = 0 at diagnosis and start treatment, *t* = 1 is 3 months after treatment, *t* = 2 is 6 months after treatment). **(e)** *Granzyme‐B* expression in RCDII cell lines P1 and P2, negative control SUDHL4 and activated CD8^+^ T cells as positive control, using RT‐MLPA analysis. **(f)** Representative histograms of granzyme‐B protein expression in RCDII cell lines P1 and P2 and control cells using flow cytometry analysis; grey shaded peak, isotype‐matched control. Cell experiments were done in triplicate. **P ≤ 0.01, ***P ≤ 0.001, unpaired *t*‐test, results shown as mean + sem.

First, *granzyme‐B* expression was analysed in 10 RCDII patients and 12 control patients with CD on GFD. mRNA expression of granzyme‐B was significantly upregulated in aberrant IEL of patients with RCDII (*P* = 0.0022, Figure 1a). However, transcripts for granzyme‐B showed a high variability amongst the RCDII patients. On protein level, intracellular granzyme‐B expression was remarkably increased in RCDII patients (*P* = 0.0001, Figure 1b). In all tested RCDII patients, a higher granzyme‐B protein expression was found compared to each CD patient on GFD.

RCDII patients display a dramatic loss of villi in the small intestine. Therefore, we investigated whether granzyme‐B protein expression levels in aberrant IEL correlate with the severity of villous atrophy in patients with RCDII after treatment. Non‐responding patients with persistent villous atrophy (Marsh stages 2 and 3) showed significantly higher granzyme‐B protein expression than responders in which full histological recovery (Marsh 0) was observed (*P* = 0.0023, Spearman Rho's correlation coefficient 0.824, Figure 1c). The correlation was independent of aberrant IEL percentage (Supplementary table 1). Interestingly, in responder RCDII patients, granzyme‐B reached completely similar expression levels after successful treatment as in CD patients on GFD. These findings suggest that granzyme‐B protein expression in aberrant IEL of RCDII patients is positively correlated to the degree of mucosal damage. Figure 1d showed follow‐up measurements of granzyme‐B protein expression in two treated RCDII patients. Both patients demonstrated high levels of granzyme‐B expression before treatment with cladribine, but only in the patient showing recovery of villous architecture (responder) a clear decrease in granzyme‐B expression was observed. In the non‐responder RCDII patient, the granzyme‐B protein level remained high. These data indicate the clinical importance of granzyme‐B as a marker in the follow‐up of RCDII patients.

High expression of both granzyme‐B mRNA and protein was observed in the RCDII‐derived cell lines P1 and P2 (Figure 1e and f). Levels of granzyme‐B expression were comparable in the RCDII cells and cytotoxic T cells.

Delivery of granzyme‐B into the target cell is dependent on the pore‐forming molecule perforin.^17^ In accordance with *granzyme‐B* expression, *perforin* expression was also markedly elevated in RCDII patients compared to CD patients on GFD (*P* = 0.0049, Supplementary figure 1A). In most RCDII patients with a relatively high *granzyme‐B* expression, a high *perforin* expression was also observed (Supplementary figure 1B). Thus, it seems that the level of *granzyme‐B* expression is related to the mRNA level of perforin. In RCDII cell lines, high levels of both mRNA and protein expression of perforin were present (Supplementary figure 1C and D).

Granzyme‐B can be inhibited by the serpin proteinase inhibitor PI‐9.^18^ mRNA and/or protein expression of PI‐9 was detected in aberrant IEL of RCDII patients and RCDII cell lines (Supplementary figure 2A–C). This PI‐9 expression is likely to protect the aberrant IEL from damage by their endogenously produced granzyme‐B.

In conclusion, granzyme‐B is significantly upregulated in aberrant IEL of RCDII patients and its expression level is positively correlated with the degree of villous atrophy.

### Granzyme‐B is secreted by aberrant IEL in the presence of enterocytes

**Figure 2:**
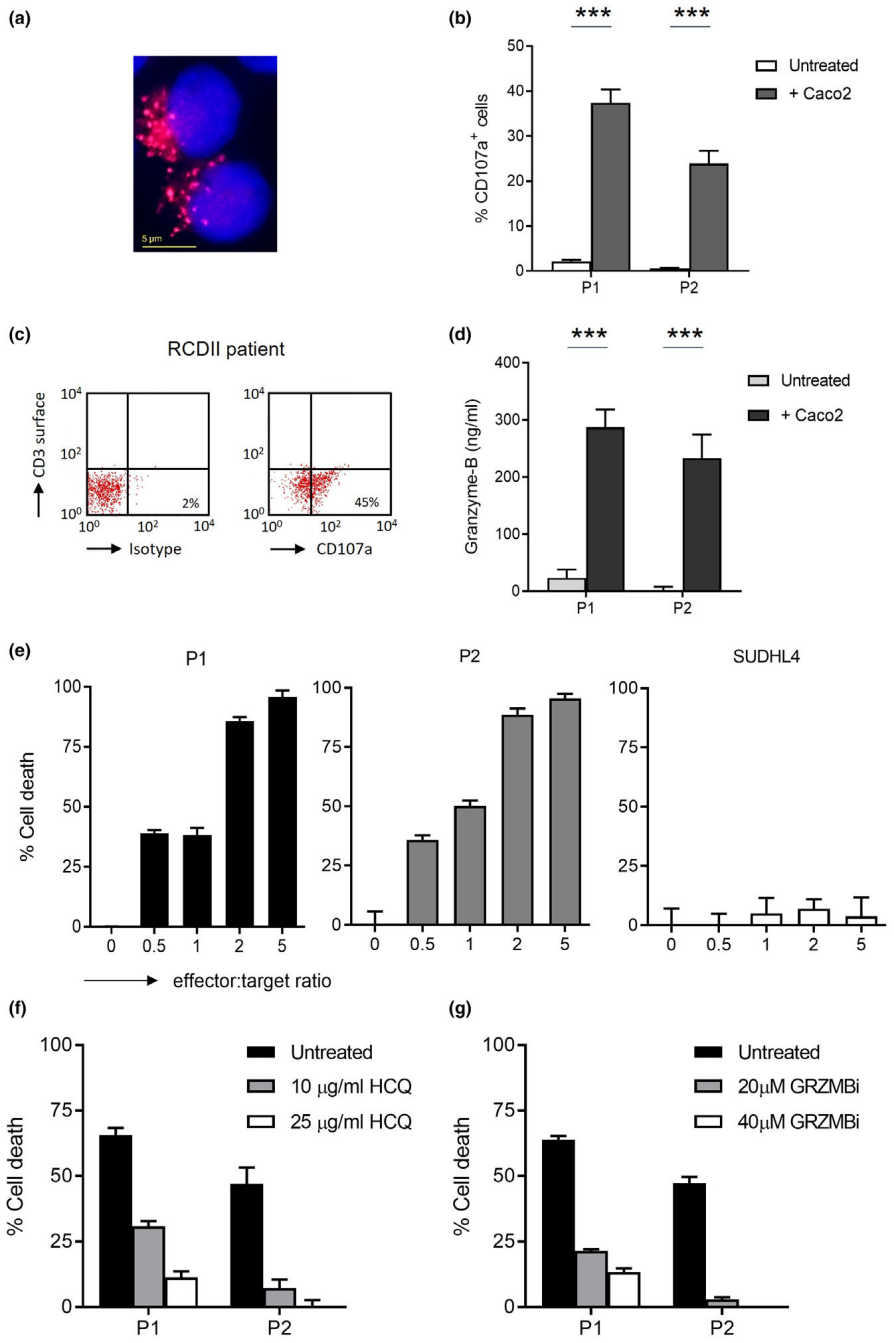
Granzyme‐B is the key cell death mediator in enterocyte‐induced cell death and is only secreted by aberrant intra‐epithelial T‐lymphocyte (IEL) in the presence of enterocytes. **(a)** Cytoplasmic localisation of granzyme‐B granules in refractory celiac disease type II (RCDII) cells P2 using immunofluorescence (red, granzyme‐B; blue, DAPI nucleus staining, 100× magnification). **(b)** Expression of degranulation marker CD107a on RCDII cell lines P1 and P2 in the absence and presence of epithelial Caco2 cells after 4 h of incubation. **(c)** CD107a expression on aberrant IEL of a duodenal biopsy from a representative RCDII patient with villous atrophy after 4 h of incubation; left panel, isotype‐matched control. **(d)** Granzyme‐B secretion by RCDII cell lines in the absence and presence of enterocyte cell line Caco2 after 6 h of incubation. **(e)** RCDII cell lines P1 and P2 induce killing of epithelial Caco2 cells. The control cell line SUDHL4 showed no cytotoxicity against the Caco2 cells. **(f)** Enterocyte cell death by RCDII cells incubated with increasing concentrations of degranulation blocker hydroxychloroquine sulphate (HCQ). **(g)** Killing of intestinal Caco2 cells by RCDII cells in the presence of increasing concentrations of the granzyme‐B inhibitor Z‐AAD‐CH2Cl. For cell death assays, cytotoxicity was measured after 16 h of co‐incubation at an effector:target ratio 2:1. Cell experiments were done in triplicate. ***P ≤ 0.001, unpaired *t*‐test, results are shown as mean + sem.

Intracellular granzyme‐B can be secreted during degranulation by cytotoxic T cells and NK cells. In RCDII cells, granzyme‐B was localised in granules throughout the cytoplasm (Figure 2a). Lytic granules containing granzyme‐B can move across the cytoplasm facilitated by CD107, a lysosome‐associated membrane‐protein that reallocates to the cell‐surface membrane during degranulation.^19^ Release of granules by RCDII cells, measured by activated CD107 (CD107a) expression, only occurred in the presence of Caco2 intestinal epithelial cells (Figure 2b). In RCDII patient biopsies that demonstrated severe villous atrophy, evident degranulation of aberrant IEL was also observed (Figure 2c). Considering granzyme‐B accumulates in granules and aberrant IEL show degranulation, the extracellular concentration of granzyme‐B was determined. As measured by ELISA, high levels of soluble granzyme‐B were detected in cell supernatants (Figure 2d). Thus, in the presence of enterocytes, aberrant IEL degranulate and subsequently secrete granzyme‐B.

### Aberrant IEL induce granzyme‐B‐mediated killing of enterocytes mainly via activation of the intrinsic apoptosis pathway

Secreted granzyme‐B is able to induce cell death, eliminating a target cell. Therefore, we examined whether intestinal epithelial Caco2 cells are killed by aberrant IEL. RCDII cells showed high levels of cytotoxicity against enterocytes. Cell death levels rose with increasing effector:target ratios, and at a ratio of 5:1 the RCDII cells killed almost all enterocytes (Figure 2e). In the absence of degranulation, enterocyte cell death was significantly reduced (Figure 2f). To confirm that enterocyte loss was induced by granzyme‐B, RCDII cells were incubated with a specific granzyme‐B inhibitor. Inhibition of granzyme‐B blocked epithelial cell killing, indicating that enterocyte cell death by aberrant IEL is granzyme‐B dependent (Figure 2g). Enterocyte killing by RCDII cell line P1 was almost completely blocked by the granzyme‐B inhibitor, but an alternative cell death inducer might also make a small contribution to cytotoxicity in this cell line. After exposure to the pancaspase inhibitor zVAD‐FMK, enterocyte cell death was evidently blocked (Supplementary figure 3A). Furthermore, granzyme‐B caused depolarisation of the mitochondrial membrane in enterocytes, and activation of caspase 3/7 and caspase 9 was observed (Supplementary figure 3B–D). Experiments with a specific caspase‐9 inhibitor LEHD‐FMK demonstrated that a small part of the detected enterocyte cell death was caspase‐9 independent, suggesting that granzyme‐B also likely induces apoptosis by direct activation of the effector caspases 3/7 (data not shown). Taken together, enterocyte killing by aberrant IEL is granzyme‐B dependent and is induced particularly via activation of the intrinsic apoptosis pathway.

### Aberrant IEL induce enterocyte killing by cell–cell binding and demonstrate upregulated expression of CD103

**Figure 3:**
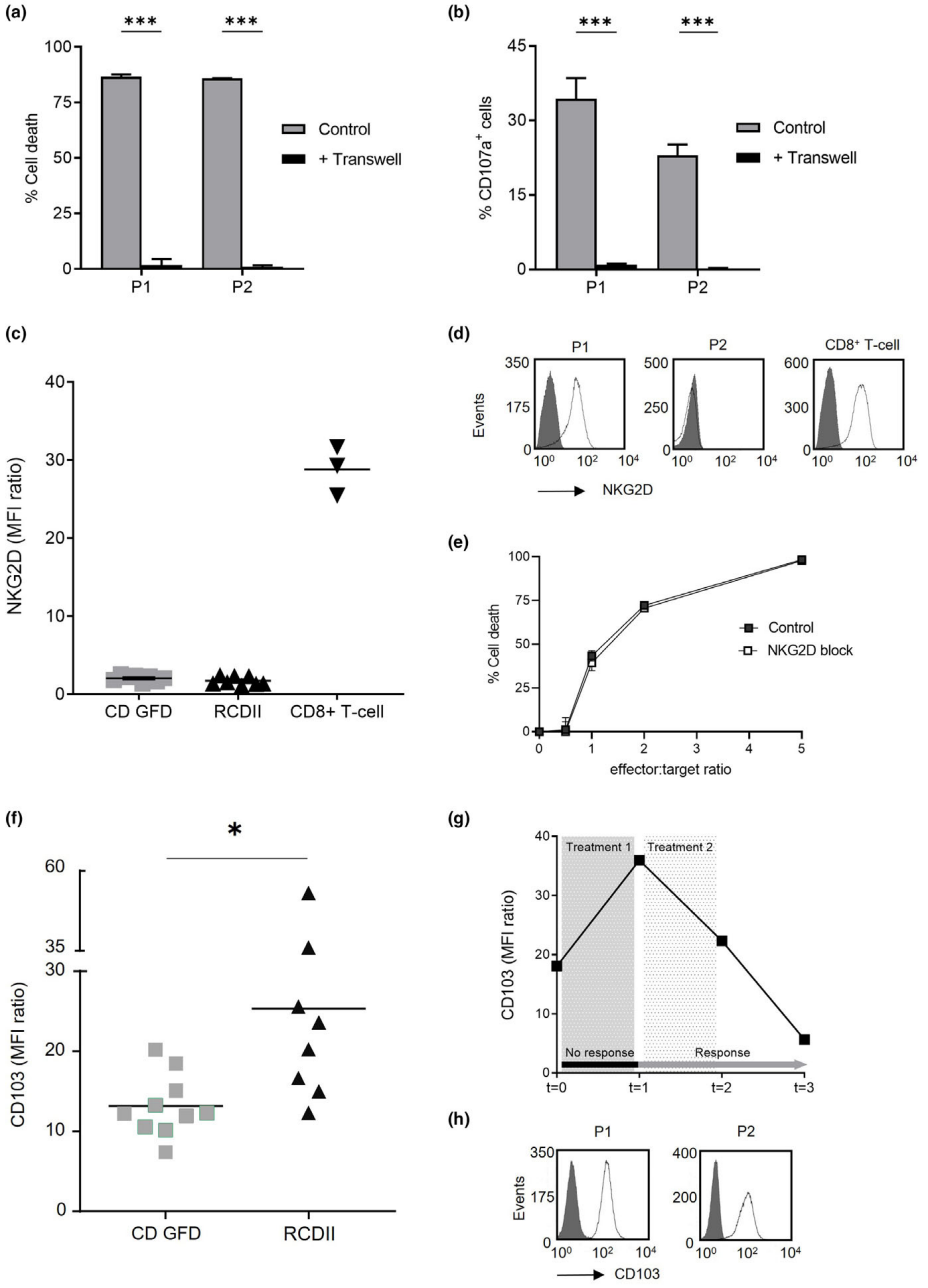
Aberrant intra‐epithelial T‐lymphocyte (IEL) demonstrate upregulated expression of CD103 and require cell–cell binding to induce enterocyte killing. **(a)** Refractory celiac disease type II (RCDII) cell‐induced cytotoxicity of Caco2 cells in the presence of a transwell system. Cell death was measured after 16 h of co‐incubation at an effector:target ratio 2:1. **(b)** Degranulation by RCDII cells in the presence of a transwell system. CD107a expression was measured after 4 h of co‐incubation with Caco2 cells at an effector:target ratio 2:1. **(c)** NKG2D expression on aberrant IEL of RCDII patients and patients with celiac disease (CD) on gluten‐free diet (GFD), using flow cytometry analysis. Activated CD8+ T cells served as positive control. **(d)** Representative histograms of NKG2D expression on RCDII cell lines P1 and P2 using flow cytometry; grey shaded peak, isotype‐matched control. **(e)** Killing of epithelial cells Caco2 by RCDII cell line P1 in the presence of 20 μg/mL NKG2D‐blocking mAb or isotype control mAb. **(f)** CD103 expression on aberrant IEL of RCDII patients and patients with CD on GFD, using flow cytometry analysis. **(g)** Follow‐up of CD103 expression on aberrant IEL from a representative RCDII patient, showing persistent villous atrophy after first‐line treatment (cladribine; non‐responding) and complete mucosal recovery after second‐line treatment (autologous stem cell transplantation; responding). *t* = 0 at diagnosis and start treatment, *t* = 1 is 3 months after first‐line treatment, *t* = 2 and *t* = 3 is 3, respectively, 6 months after stem cell transplantation. **(h)** Histograms of CD103 expression on RCDII cell lines P1 and P2 using flow cytometry analysis; grey shaded peak, isotype‐matched control. Cell experiments were done in triplicate. *P ≤ 0.05, ***P ≤ 0.001, unpaired *t*‐test, results shown as mean + sem.

Enterocyte cell death was caused by degranulation of granzyme‐B; however, aberrant IEL only secreted granzyme‐B in the proximity of intestinal epithelial cells. In order to investigate whether direct binding of aberrant IEL to enterocytes is necessary to induce degranulation, transwell assays were performed. The transwell prevented physical binding between lymphocyte and enterocyte but allowed exchange of soluble extracellular factors. In the presence of a transwell, RCDII cells completely lost their cytotoxic effects on enterocytes and no cell death was observed (Figure 3a). Accordingly, degranulation of RCDII cells did not occur when IEL and enterocytes were physically separated (Figure 3b). These figures illustrate that degranulation and subsequent enterocyte killing by aberrant IEL is completely dependent on direct cell–cell binding.

Next, we investigated possible receptor‐ligand interactions required for granzyme‐B induced enterocyte cell death. Previously, it was shown that the NKG2D‐MICA binding activates TCR‐independent CD8+ IEL cytotoxicity in active CD.^20^ To study whether in RCDII aberrant IEL kill enterocytes via NKG2D signalling, expression levels of NKG2D were analysed in patient‐derived duodenal biopsies. Little or no surface expression of NKG2D was found on aberrant IEL of RCDII patients, similar to expression levels observed in CD patients on GFD (Figure 3c). RCDII‐derived cell line P2 lacked NKG2D expression; however, in RCDII cell line P1, expression of NKG2D was detected (Figure 3d). Nonetheless, blocking of NKG2D on RCDII cells P1 did not inhibit any enterocyte killing (Figure 3e). These findings support that NKG2D is not involved in the cell death of the gut epithelium in RCDII.

Aberrant IEL are characterised by constitutive expression of the receptor CD103, also known as αE integrin. Binding of CD103 to its ligand was previously found to trigger lytic granule polarisation and subsequent exocytosis by NK cells.^21^ Therefore, we examined whether CD103 is important for degranulation and enterocyte killing by aberrant IEL. Expression of CD103 was increased on the surface of aberrant IEL of RCDII patients, compared to CD patients on GFD (*P* = 0.0146, Figure 3f). CD103 was heterogeneously expressed in RCDII patients. No clear correlation was found between CD103 and granzyme‐B expression (data not shown). In Figure 3g, CD103 expression was monitored at an intra‐individual level in a representative newly diagnosed RCDII patient. An increase of CD103 expression was observed after failure of first‐line treatment with cladribine; however, after successful second‐line therapy with autologous stem cell transplantation, CD103 expression strongly decreased. Both RCDII cell lines clearly presented CD103 on their membrane (Figure 3h).

### Aberrant IEL‐enterocyte binding via CD103 induces granzyme‐B‐mediated enterocyte cell death

**Figure 4:**
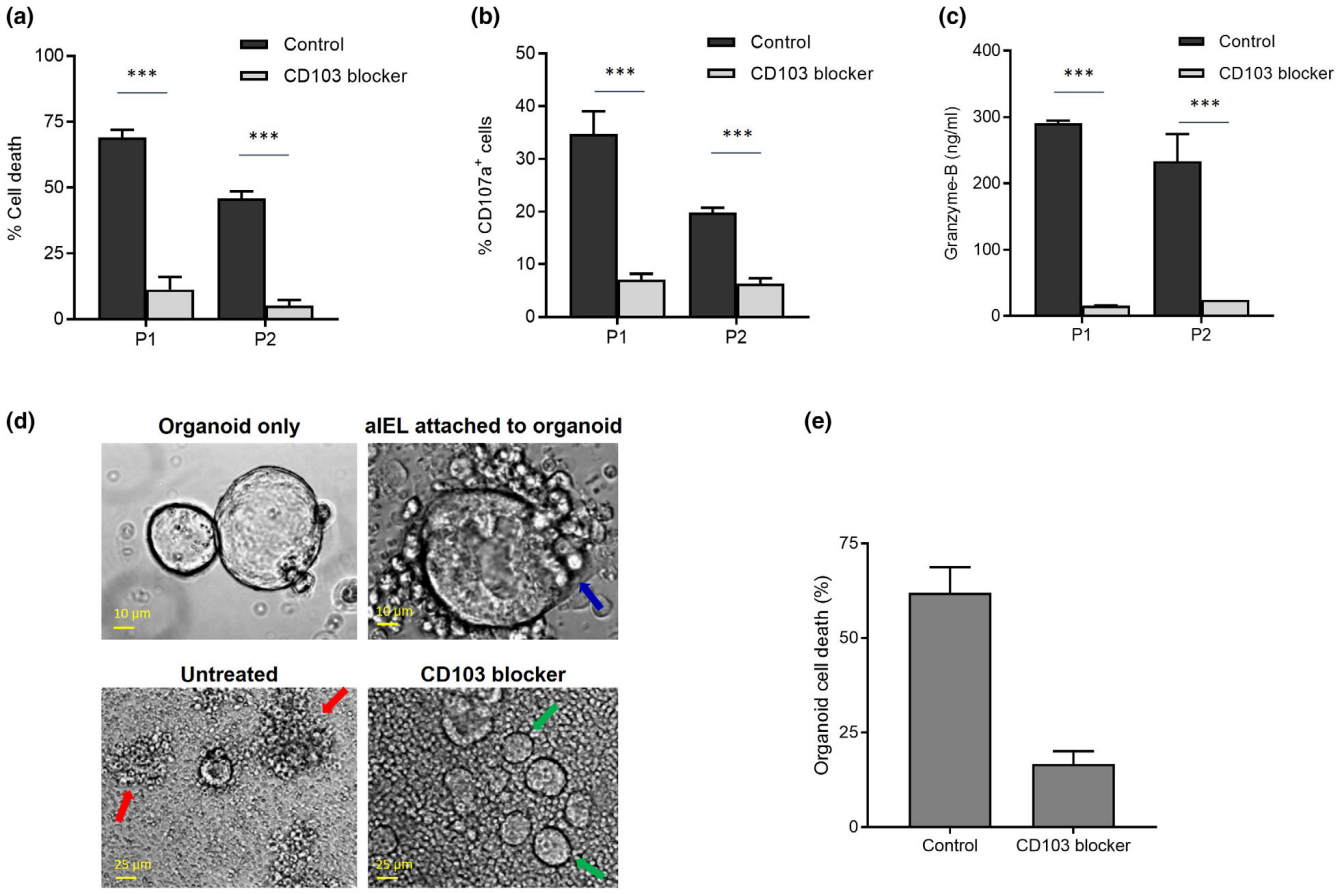
Aberrant intra‐epithelial T‐lymphocyte (IEL)‐enterocyte binding via CD103 induces granzyme‐B‐mediated enterocyte cell death. **(a)** Killing of Caco2 epithelial cells by refractory celiac disease type II (RCDII) cell lines in the presence of 10 μg/mL CD103‐blocking mAb or isotype control. Cell death was measured after 16 h of co‐incubation at an effector:target ratio 2:1. **(b)** Degranulation by RCDII cell lines, measured by CD107a expression, in the presence of 10 μg/mL CD103‐blocking mAb or the matching isotype control. Degranulation was measured after 4 h of co‐incubation with Caco2 cells at an effector:target ratio 2:1. **(c)** Secretion of granzyme‐B by RCDII cell lines co‐incubated with Caco2 cells in the presence of 10 μg/mL CD103‐blocking mAb compared to the isotype control. Secretion was measured after 6 h of co‐incubation at an effector:target ratio 2:1. **(d)** Upper left picture: small intestinal organoids; upper right picture: attachment of RCDII cells to an organoid (blue arrow); lower left picture: RCDII cells induce killing of organoids (red arrows); lower right picture: in the presence of 10 μg/mL CD103‐blocking mAb RCDII–induced organoid cell death is evidently reduced, illustrated by the presence of viable organoids (green arrows). **(e)** Induction of organoid cell death by RCDII cells P2 in the presence of 10 μg/mL CD103‐blocking antibody or an isotype control, measured by cell count via microscopy. Killing was measured after 24 h of co‐incubation at an effector:target ratio 50:1. Figure 4d, upper pictures 25× magnification, lower pictures 10× magnification, using an Olympus microscope. Cell experiments for Figure 4e performed in duplicate, other cell experiments performed in triplicate. ***P ≤ 0.001, unpaired *t*‐test, results shown as mean + sem.

Subsequently, we investigated whether CD103 is necessary for induction of granzyme‐B‐mediated enterocyte killing by the aberrant IEL. CD103‐mediated release of granzyme‐B was previously described.^22^ Blocking of the CD103 receptor significantly inhibited cytotoxicity against enterocytes (Figure 4a). Moreover, degranulation by RCDII cells was strongly reduced in the presence of a CD103‐blocker (Figure 4b). Secretion of granzyme‐B was also diminished if CD103‐mediated binding was blocked (Figure 4c). Taken together, CD103 expressed on aberrant IEL is essential for granzyme‐B‐mediated enterocyte killing in RCDII.

These findings were additionally tested using human intestinal organoids. IEL can be maintained in co‐culture with organoids, providing a three‐dimensional system mimicking the *in vivo* situation in which migration, binding and the induced cell death process can be visualised.^23^ During the first 4 h of co‐incubation, migration of aberrant IEL to the organoids was observed. Consecutively, the aberrant IEL attached to the surface of the organoids (Figure 4d, upper right panel, blue arrow). Binding of the aberrant IEL resulted in killing of the organoid cells, which was characterised by a process of disintegration and falling apart of the organoids. Approximately 60% of organoid cells were killed after 24 h of co‐incubation (Figure 4d, lower left panel, red arrows). In the presence of the CD103‐blocker, intestinal cell death was significantly inhibited and the organoids remained intact (Figure 4d, lower right panel, green arrows; Figure 4e). The viable organoid cells had a similar morphology to organoid cells cultured without aberrant IEL (Figure 4d, upper left panel). These findings further support that aberrant IEL cause loss of villi via CD103.

### Granzyme‐B‐mediated killing of enterocytes is inhibited by etrolizumab

**Figure 5:**
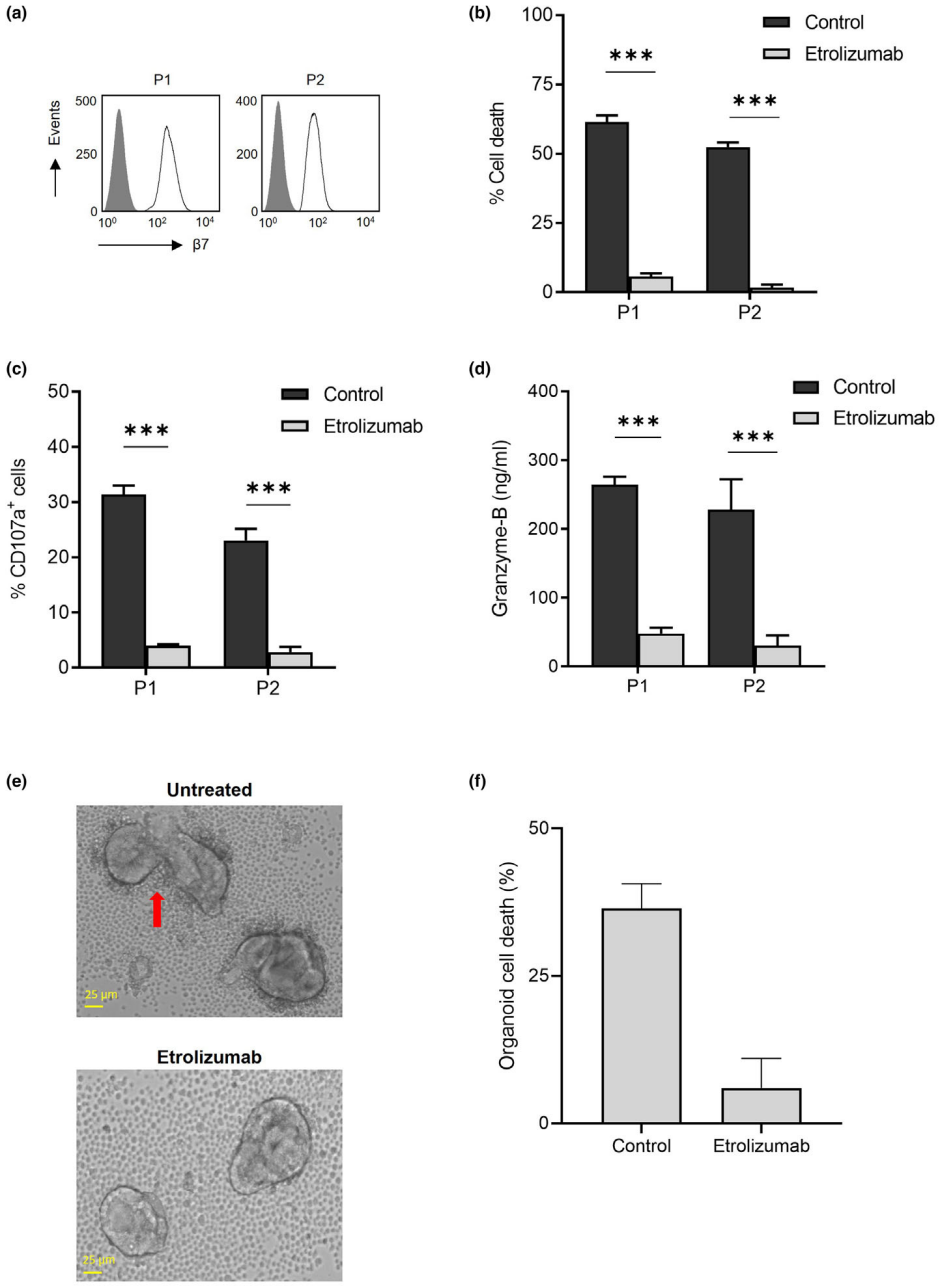
Etrolizumab inhibits granzyme‐B secretion and restores enterocyte viability. **(a)** Histograms of β7 expression on refractory celiac disease type II (RCDII) cell lines P1 and P2; grey shaded peak, isotype‐matched control. **(b)** Caco2 cell death by RCDII cells in the presence of 50 μg/mL etrolizumab or isotype control. Cell death was determined after 16 h of co‐incubation at an effector:target ratio 2:1. **(c)** Degranulation by RCDII cell lines in the presence of 50 μg/mL etrolizumab or the matching isotype control. Degranulation was measured after 4 h of co‐incubation with Caco2 cells at an effector:target ratio 2:1. **(d)** Secretion of granzyme‐B by RCDII cell lines co‐incubated with enterocyte Caco2 cells in the presence of 50 μg/mL etrolizumab compared to the isotype control. Secretion was measured after 6 h of co‐incubation at an effector:target ratio 2:1. **(e)** Upper picture: RCDII cells attach to the organoid surface and cause membrane disruption (red arrow), inducing organoid cell death; lower picture: the attachment of RCDII cells to organoids and subsequent organoid cell death is decreased in the presence of 50 μg/mL etrolizumab antibody. **(f)** Induction of organoid cell death by RCDII cells P2 in the presence of 50 μg/mL etrolizumab or an isotype control, measured by cell count via microscopy. Killing was measured after 24 h of co‐incubation at an effector:target ratio 20:1. Figure 5e, pictures 10× magnification, using an Olympus microscope. Cell experiments were done in triplicate. ***P ≤ 0.001, unpaired *t*‐test, results shown as mean + sem.

CD103 usually forms a heterodimeric complex with integrin β7, which is present on aberrant IEL. In RCDII cell lines P1 and P2, β7 is expressed on the cell surface (Figure 5a). β7 can be blocked by the humanised monoclonal antibody etrolizumab. Incubation of RCDII cells with etrolizumab caused a significant decrease in enterocyte cell death (Figure 5b). Almost no killing of epithelial cells was observed. Furthermore, functional inhibition of β7 by etrolizumab evidently decreased degranulation and subsequent granzyme‐B secretion by RCDII cells (Figure 5c and d). These data demonstrate that granzyme‐B‐mediated enterocyte killing by aberrant IEL can be blocked by etrolizumab.

### Treatment with etrolizumab blocks induction of intestinal epithelial damage by aberrant IEL in a preclinical human organoid model

**Figure 6:**
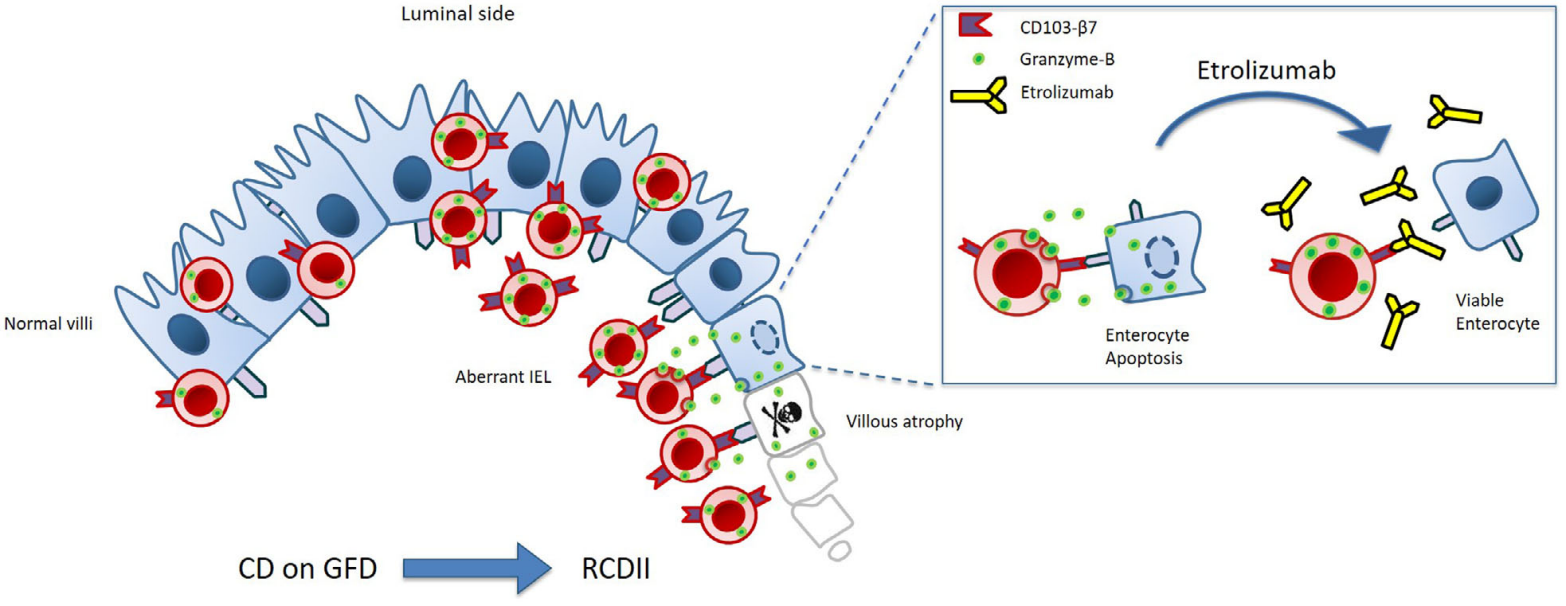
Targeting the cell death mechanism responsible for villous atrophy in refractory celiac disease type II (RCDII). Aberrant intra‐epithelial T‐lymphocyte (IEL) cause mucosal damage in RCDII by granzyme‐B‐induced apoptosis of enterocytes through CD103‐β7 binding. Treatment with anti‐β7 monoclonal antibody etrolizumab inhibits granzyme‐B‐induced enterocyte killing and restores the viability of the intestinal mucosa.

Etrolizumab is currently explored as a treatment option for patients with inflammatory bowel disease.^24^, ^25^ To further investigate the potential of etrolizumab as a novel therapy for RCDII, we determined its efficacy in our preclinical human intestinal organoid model. In the presence of an isotype control antibody, RCDII cells attached to the surface of the organoids and caused disruption of the membrane integrity leading to intestinal cell death (Figure 5e, upper panel, red arrow). Treatment with etrolizumab resulted in lack of binding of aberrant IEL to organoids (Figure 5e, lower panel). Killing of enterocytes by RCDII cells was prevented and a reduction of epithelial damage was observed (Figure 5f). In the presence of etrolizumab, organoids remained intact and viable. In conclusion, targeting the aberrant IEL with etrolizumab inhibits intestinal epithelial killing and therefore etrolizumab might be a potential treatment to restore villous structure in RCDII (Figure 6, graphical abstract).

## Discussion

Severe enteropathy is one of the key features of RCDII, resulting in high morbidity and poor clinical outcome. The extensive mucosal damage found in RCDII is induced by aberrant IEL.^12^ However, the cell death mechanism leading to villous atrophy is unclear. In this study, we investigated how aberrant IEL induce killing of enterocytes in RCDII.

mRNA and protein expression of granzyme‐B were strongly upregulated in aberrant IEL of RCDII patients. Our data demonstrated that aberrant IEL released granzyme‐B in the presence of intestinal epithelial cells. Once in enterocytes, granzyme‐B caused cell death by direct activation of caspase 3/7 and/or via triggering of the intrinsic apoptosis pathway, which is in agreement with previous studies in cancer.^26^ Functional analyses revealed that granzyme‐B was the most important cell death mediator of enterocyte cell death in RCDII.

Previous studies demonstrated that upregulated granzyme‐B in RCDII can cleave the transmembrane receptor NOTCH1 into a dysfunctional protein lacking transcriptional activity.^27^, ^28^ This process results in dedifferentiation of these mature lymphocytes into aberrant IEL, emphasising the significance of granzyme‐B in the pathogenesis of RCDII. The observed increase in granzyme‐B expression might be caused by IL‐15, which is overexpressed in the intestinal epithelium of RCDII patients as was previously shown.^15^, ^28^ IL‐15 phosphorylates transcription factor STAT5. Activated STAT5 binds to the promoter region of granzyme‐B, strongly driving the transcription of granzyme‐B.^29^, ^30^

The level of granzyme‐B protein expression in aberrant IEL of RCDII patients correlated with the severity of villous atrophy, indicating that granzyme‐B can be a marker for disease activity. Moreover, in the follow‐up of individual RCDII patients, granzyme‐B was also a promising marker as the change in its expression level corresponded with response to therapy. The clinical utility of granzyme‐B monitoring needs to be further confirmed in prospective studies. Previous data in serum showed that the soluble granzyme‐B concentration in RCDII patients was evidently higher than in CD patients on GFD.^16^ These findings might reflect the disease activity in the celiac intestine and therefore future studies should also elucidate if granzyme‐B can be used as a serological biomarker for disease activity.

We demonstrated that for degranulation and secretion of granzyme‐B, cell–cell binding between the aberrant IEL and enterocyte was necessary. The CD103(αE) receptor is an integrin expressed on IEL that is responsible for gut specific T‐cell homing by recruiting these lymphocytes to mucosal surfaces.^31^ In RCDII patients, expression of CD103 on aberrant IEL was upregulated. CD103 forms together with the subunit β7 a complex, which binds to its principal ligand E‐cadherin found on epithelial cells.^32^ We also found significant upregulation of E‐cadherin protein expression on enterocytes of RCDII patients (Supplementary figure 4). Expression of CD103 is mainly induced by the cytokine transforming growth factor‐β1 (TGF‐β1) and furthermore, TGF‐β1 enhances the affinity of CD103 for E‐cadherin.^33^ The cause of increased CD103 expression in RCDII is unclear and requires further research, as in a previous study comparing RCDII and CD on GFD patients, the intestinal protein expression of TGF‐β1 was not different.^34^ Our current study revealed that CD103‐mediated binding between RCDII cells and enterocytes was essential for degranulation of granzyme‐B and subsequent enterocyte killing. Interaction with CD103 can activate phospholipase Cγ, leading to intracellular reorganisation of the microtubules along which granzyme‐B granules are transported. Granzyme‐B then relocalises to the cell membrane, followed by exocytosis in the presence of a target cell.^21^, ^35^ Thus in RCDII, CD103 might not only act as an integrin but could also directly mediate granzyme‐B dependent enterocyte cell death, ultimately leading to severe mucosal damage. In agreement, it was recently shown in a mouse model that intra‐epithelial γδ T cells can induce enterocyte cell death via CD103‐mediated granzyme release.^22^

In the absence of other suitable preclinical models of RCDII, we also demonstrated the functional and therapeutic significance of the CD103‐receptor using human small intestinal organoids in which IEL migration and spatial interaction between aberrant IEL and enterocytes can be visualised. In this new three‐dimensional model, RCDII cells migrated towards the organoids and induced CD103‐dependent killing of the organoids. Novel insights in the important interplay between aberrant IEL and enterocytes in RCDII could be further provided by organoid studies.

In RCDII, an additional receptor in the TCR‐independent killing process could be present. NKG2D was hardly expressed on aberrant IEL in RCDII patients, similar to findings of a previous study.^36^ This suggests that NKG2D does not contribute to the severe mucosal damage in RCDII, in contrast with its important role in active CD. Other receptors described to be involved in TCR‐independent granzyme‐B‐mediated cytotoxicity include the cation‐independent mannose 6‐phosphate receptor (CI‐M6PR) and CD44.^37^, ^38^ However, in RCDII patients, enterocyte expression of both CI‐M6P and CD44 was not present (data not shown). Another potential candidate could be NKp46, as it is highly and selectively expressed on aberrant IEL of RCDII patients and capable of inducing degranulation when triggered by its ligand.^39^, ^40^

Current treatments for patients with RCD are often ineffective resulting in a poor prognosis. Our results provide new leads for targeted therapy in RCDII. Etrolizumab, which targets β7, demonstrated remarkable reduction of the cytotoxic effects of aberrant IEL against enterocytes. The efficacy of etrolizumab can be explained by the strong decrease in degranulation and secretion of granzyme‐B by aberrant IEL. Moreover, in the preclinical organoid model, etrolizumab prevented intestinal epithelial cell killing, leading to survival of the organoids. Etrolizumab is currently investigated as potential therapy in IBD and showed a strong safety profile.^24^, ^25^ Furthermore, especially in IBD patients with high granzyme expression, etrolizumab was effective.^24^ Taken together, these results indicate that etrolizumab is a rational candidate drug for treatment of villous atrophy in RCDII.

We conclude that killing of enterocytes in RCDII depends on degranulation of granzyme‐B by aberrant IEL through CD103‐β7‐binding. Targeting the CD103‐β7 complex by etrolizumab inhibited granzyme‐B‐mediated enterocyte cell death. These findings contribute to a better understanding of the pathogenesis of villous atrophy in RCDII and are important for diagnostic purposes. Furthermore, our preclinical data identified etrolizumab as a promising therapy for patients with RCDII, indicating that etrolizumab should be further evaluated in clinical studies.

## Methods

All authors had access to the study data and have reviewed and approved the final manuscript. The data sets generated and/or analysed during this study will be available from the corresponding author upon reasonable request.

### Patient selection

Adult patients with RCDII or CD on GFD (controls) were included between 2009 and 2018. No included patients developed an EATL. CD was diagnosed according to current guidelines, that is if duodenal biopsies showed increased numbers of IEL, crypt hyperplasia and/or villous atrophy together with positive celiac specific serology (antibodies against transglutaminase 2, endomysium, and/or deamidated gliadin).^41^ CD patients on GFD were incorporated if follow‐up duodenal biopsies showed normalisation of villus architecture, < 20% aberrant IEL and negative celiac specific serology. RCDII patients were included based on persisting or recurring malabsorption symptoms, histological abnormalities (Marsh stages 1–3) and ≥ 20% aberrant IEL despite strict adherence to a GFD reflected in negative serology. Responder RCDII patients were defined by full histological recovery of the intestine after treatment, while non‐responder RCDII patients showed persistent villous atrophy. Patient characteristics for all patients included in this study are demonstrated in Supplementary table 1.

### Human materials

During upper gastrointestinal endoscopy, large spike forceps biopsies (MediGlobe) were taken of the second part of the duodenum for diagnostic purposes. All protocols for obtaining and studying biopsy specimens and patients' data were approved within the local ethical procedures of the institutional ethical review board at the Amsterdam UMC, location VU University Medical Center and complied with the Code of Conduct for Medical Research in The Netherlands (www.federa.org). IEL single‐cell suspensions were isolated from duodenal biopsies as previously described.^42^, ^43^ Aberrant IEL were defined as cytoplasmic CD3^+^, surface CD45^+^, CD7^+^, surface CD3^−^, CD4^−^, CD8^−^, CD16^−^ and CD56^−^. Enterocytes were identified based on the presence of epithelial cell adhesion molecule (EpCAM), absent CD45 expression and high forward and sideward scatter using FACS analysis (BD Biosciences). Human intestinal stem cells (ISC) were isolated and plated as described previously.^44^, ^45^, ^46^

### Cell culture

Human ISCs were cultured in Matrigel (growth factor reduced; BD Biosciences), supplemented with advanced DMEM/F12 containing: in‐house WNT conditioned medium (CM), R‐spondin‐1‐CM, Noggin‐CM, B‐27 (Invitrogen), recombinant EGF (Preprotech), p38i (Sigma), A38‐01 (Tocris), PGE2 (Tocris), HEPES (Sigma), N‐acetyl‐L‐cysteine (Sigma) and nicotinamide (Sigma). In order to obtain Matrigel free intestinal organoids, cultures were gently resuspended in ice cold PBS for 10 min.

RCDII cell lines P1 and P2 were established as previously described.^47^ Cells were cultured in IMDM (Gibco), supplemented with 10% FBS and 10 ng/mL IL‐15 (R&D Systems Europe) at 37°C. Restimulation was performed approximately every 4–5 weeks with 1 μg/mL phytohemagglutinin, 10 ng/mL IL‐15 and 1 × 10^6^/mL irradiated allogenous PBMCs as feeder cells. The purity of the cells was determined regularly by flow cytometry.

The intestinal epithelial cell line Caco2 was obtained from the American Type Culture Collection (ATCC) and cultured with DMEM medium (BioWhittaker) containing 10% FBS (GE Healthcare Life Sciences) and 100 IU penicillin/100 μg/mL streptomycin (1% P/S) at 37°C.

The control B‐cell lymphoma cell line SUDHL4 was obtained from the Deutsche Sammlung von Mikroorganismen und Zellkulturen GmbH (DSMZ), and cells were cultured in RPMI 1640 (BioWhittaker) with 25 mM Hepes supplemented with 10% FBS and 1% P/S. Control CD8^+^ T cells were isolated and stimulated from PBMCs, as described previously.^48^

### RT‐MLPA analysis

Aberrant IEL were sorted and isolated based on their phenotype using FACSAria (BD Biosciences). RNA was prepared of aberrant IEL from CD patients on GFD and RCDII patients as well as RCDII cell lines and controls using RNABee solution (Tel‐test Inc.). reverse transcriptase‐multiplex ligation‐dependent probe amplification (RT‐MLPA) was performed on total RNA as previously described.^49^, ^50^ Data were analysed using the GeneMapper (Applied Biosystems) and the Coffalyzer (MRC‐Holland) software. The β‐glucuronidase (GUS‐B) housekeeping gene was used to correct for effects of unequal amounts of mRNA.

### Antibodies

Cells were stained with the following antibodies: CD3, CD4, CD7, CD8, CD45, CD16 and CD56 (all BD Biosciences), CD103 (IQ Products), CD107a (BD Pharmingen), NKG2D (R&D systems Europe) and granzyme‐B (Sanquin). For intracellular granzyme‐B and CD3 detection, cells were permeabilised using Cytofix/Cytoperm and subsequently washed twice with Perm/Wash (both BD Biosciences). Cells were stained with the labelled antibodies for 30 min at 4°C. Fluorescence was detected by the FACSCalibur (BD Biosciences), and data were analysed using CellQuest (BD Biosciences).

For blocking experiments, cells were preincubated with anti‐NKG2D (20 μg/mL; R&D systems), anti‐CD103 (10 μg/mL, Beckman Coulter) or etrolisumab (50 μg/mL; Genentech) for 1 h.

### Cell death assay

Caco2 target cells were plated in a 24‐well plate at 0.6 × 10^6^/mL for 24 h, after which the cells were co‐incubated with the RCDII cell lines P1 or P2 (effector cells) at effector:target ratios between 0:1 and 5:1. Caco2 cells were incubated with increasing concentrations of the caspase‐9 inhibitor Z‐LEHD‐FMK or the pancaspase inhibitor Z‐VAD‐FMK (both Enzo Life Sciences) for 1 h prior to co‐incubation. Additionally, cellular binding between Caco2 cells and RCDII cells was prevented by a transwell system of 0.4 μm (Corning). RCDII cells were preincubated with the granzyme‐B‐inhibitor Z‐AAD‐CH2Cl (Abcam Chemicals) or the degranulation inhibitor hydroxychloroquine sulphate (Sigma‐Aldrich) for 1 h before co‐culture with the target cells. After co‐incubation at 37°C, RCDII cells were removed and Caco2 cells were washed twice with PBS. Subsequently, Caco2 cells were trypsinised, washed and kept on ice in 4 mM EDTA (Merck) to prevent cohesion. Enterocyte cell death was measured using a standard number of fluorescent beads (Fluorospheres; BD Biosciences) in combination with 7‐aminoactinomycin D (ViaProbe; BD PharMingen). Fluorescence was detected by FACSCalibur flow cytometer and analysed using CellQuest software (both BD Biosciences).

Human organoid cells were co‐incubated with RCDII cells for 24 h at 37°C. Cell death was quantified by counting all living organoids in each co‐culture by two independent expert researchers using light microscopy. Images were taken with an Olympus (CKX41; Tokyo, Japan) microscope with a Coolsnap CF camera (Photometrics, Tucson, AZ).

### ELISA

The granzyme‐B concentration was measured according to the manufacturer's instructions (Granzyme‐B ELISA Kit; Diaclone). RCDII cells and Caco2 cells were co‐incubated at an effector:target ratio of 2:1 for 6 h at 37°C, and the granzyme‐B concentration was determined in the supernatant.

### Immunocytochemistry

Cytospin preparations of RCDII cell line P2 were incubated with a directly labelled antibody against granzyme‐B (Sanquin) for 30 min at 4°C. Cells were fixed with 4% paraformaldehyde for 15 min at 37°C. DAPI was used to stain nuclei. Images were taken with a fluorescence microscope (LeicaDM4000B; Leica Microsystems).

### Caspase‐9 and caspase‐3/7 activity assays

Caspase‐9 and caspase‐3/7 activity were determined according to manufacturer's instructions (CaspaTag *in situ* assay kits; Merck Millipore). Briefly, Caco2 cells were incubated with 30x FLICA reagent dilution FAM‐DEVD‐FMK (caspase‐3/7) or FAM‐LEHD‐FMK (caspase‐9) for 1 h at 37°C. Cells were washed, and fluorescence was measured by flow cytometry.

### ΔΨm measurement

Depolarisation of the mitochondrial membrane (Ψm) in Caco2 cells was detected using the fluorescent probe tetramethylrhodamine ethyl ester perchlorate (TMRE; Invitrogen) that accumulates in mitochondria. Caco2 cells were incubated with 25 nM TMRE at 37°C for 15 min in the dark and analysed using flow cytometry. Mitochondrial membrane depolarisation was observed as a shift in the emission spectra. ΔΨm was defined as the difference in percentage unstained Caco2 cells in the presence and absence of the RCDII cell lines.

### Statistical analysis

Statistical analysis was performed using the GraphPad Prism Software. Statistical differences were determined with the unpaired Student's *t*‐test. Correlation coefficient was calculated using Spearman's Rho test. Error bars represented indicate standard error from the mean. For all experiments, significance was defined as *P ≤ 0.05; **P ≤ 0.01; ***P ≤ 0.001; ns, not significant.

## Author contributions

**Jolanda M W van de Water:** Conceptualization; investigation; validation; writing – review and editing. **Nikita Alberts:** Investigation. **Michelle van Leeuwen:** Investigation. **Laura R de Baaij:** Investigation; writing – review and editing. **Sander P J Joosten:** Investigation; resources; validation; writing – review and editing. **Petra Bonnet:** Investigation. **Yvonne M C Kooy‐Winkelaar:** Resources. **Margherita Rossi:** Investigation. **Bridget S McGlinchy:** Investigation. **Jolien C Hollander:** Investigation. **Daan A R Castelijn:** Conceptualization; data curation; formal analysis; investigation; methodology; validation; visualization; writing – original draft; writing – review and editing; project administration. **Frits Koning:** Writing – review and editing; resources. **Steven T Pals:** Writing – review and editing. **Gerd Bouma:** Writing – review and editing. **Hetty J Bontkes:** Resources; writing – review and editing. **Chris J L M Meijer:** Writing – review and editing. **Martine Reijm:** Investigation. **Chris J J Mulder:** Resources; writing – review and editing. **Saskia A G M Cillessen:** Conceptualization; investigation; data curation; methodology; resources; supervision; validation; visualization; writing – review and editing; writing – original draft; project administration.

## Conflict of interest

The authors declare no conflict of interest.

## Supporting information


Supplementary figure 1

Supplementary figure 2

Supplementary figure 3

Supplementary figure 4

Supplementary table 1

## Data Availability

The data that support the findings of this study are available from the corresponding author upon reasonable request.
