# Supplementary figures and images for: Aberrant intra‐epithelial lymphocytes cause enterocyte cell death in refractory celiac disease by CD103‐β7‐receptor‐mediated granzyme‐B degranulation which can be restored by etrolizumab
Source: Clin Transl Immunology. 2026 May 14;15(5):e70099. doi: 10.1002/cti2.70099 (PMC13175919; doi:10.1002/cti2.70099)

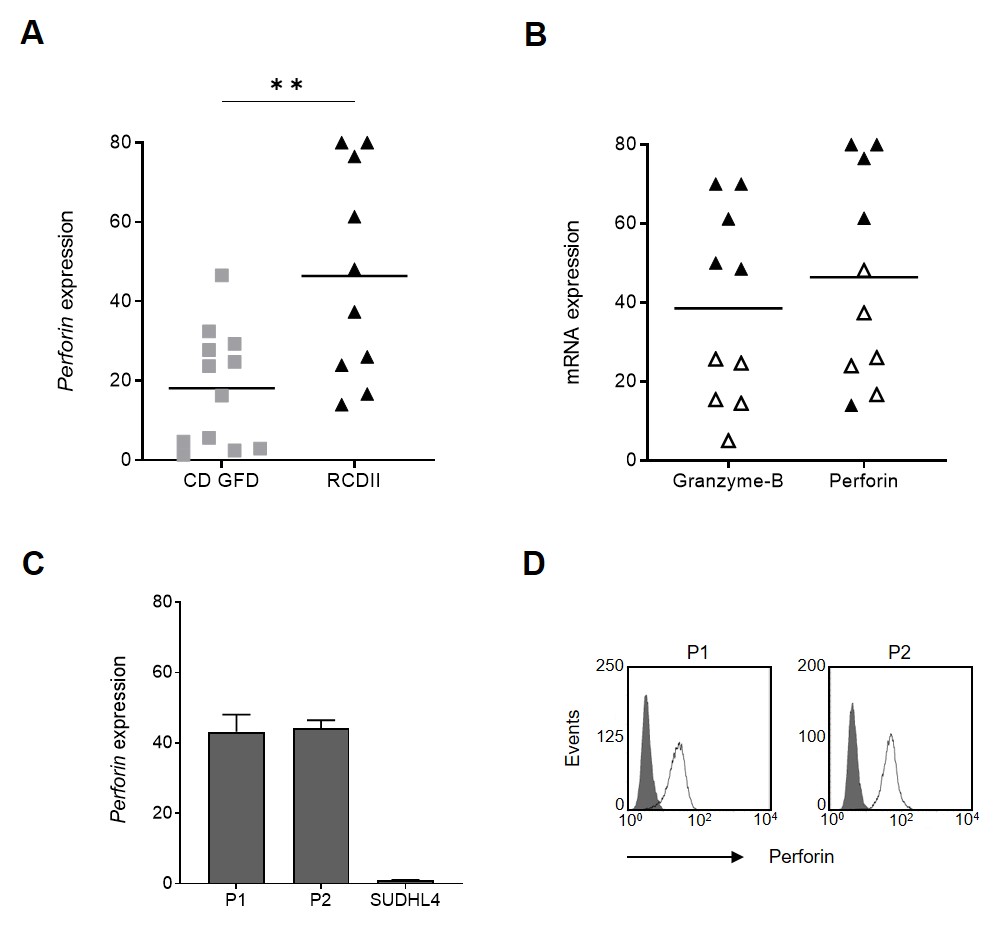

Supplement: Supplementary file 1 — Supplementary figure 1 [file CTI2-15-e70099-s004.jpg]

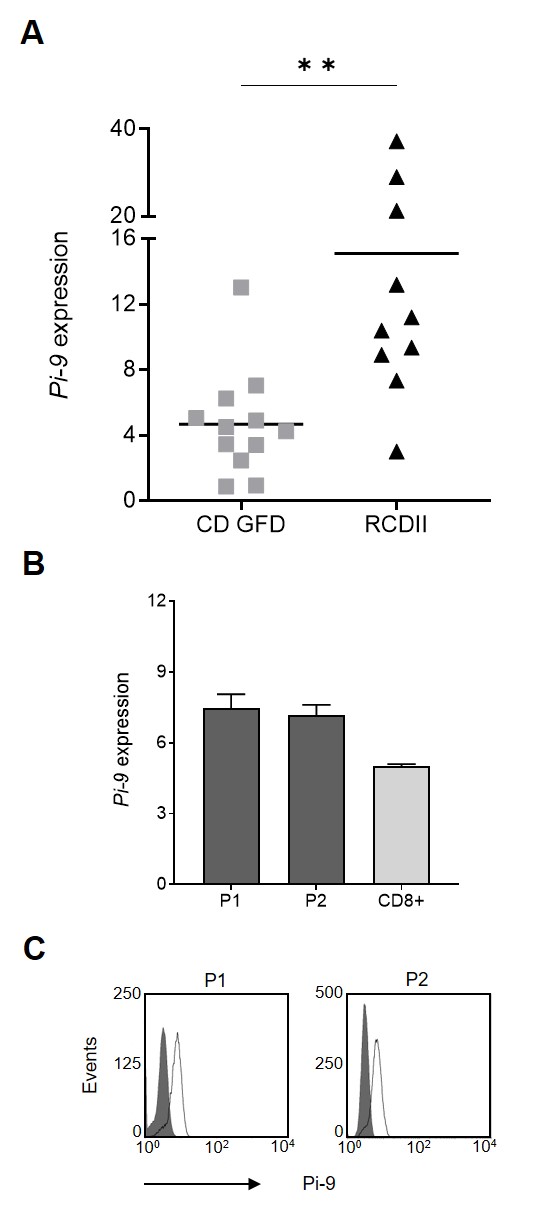

Supplement: Supplementary file 2 — Supplementary figure 2 [file CTI2-15-e70099-s003.jpg]

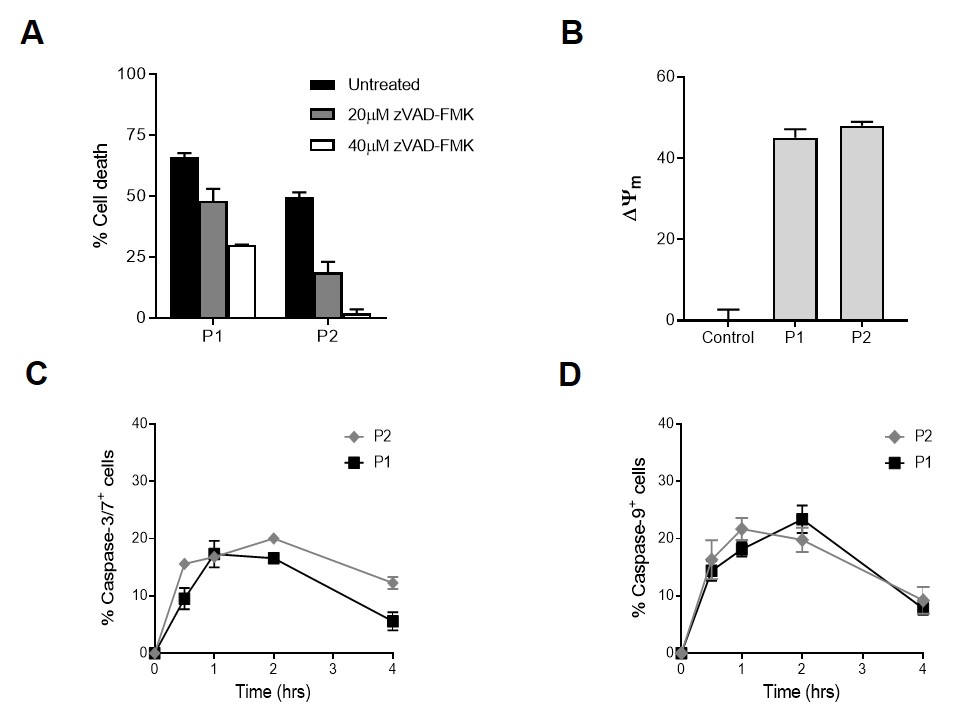

Supplement: Supplementary file 3 — Supplementary figure 3 [file CTI2-15-e70099-s001.jpg]

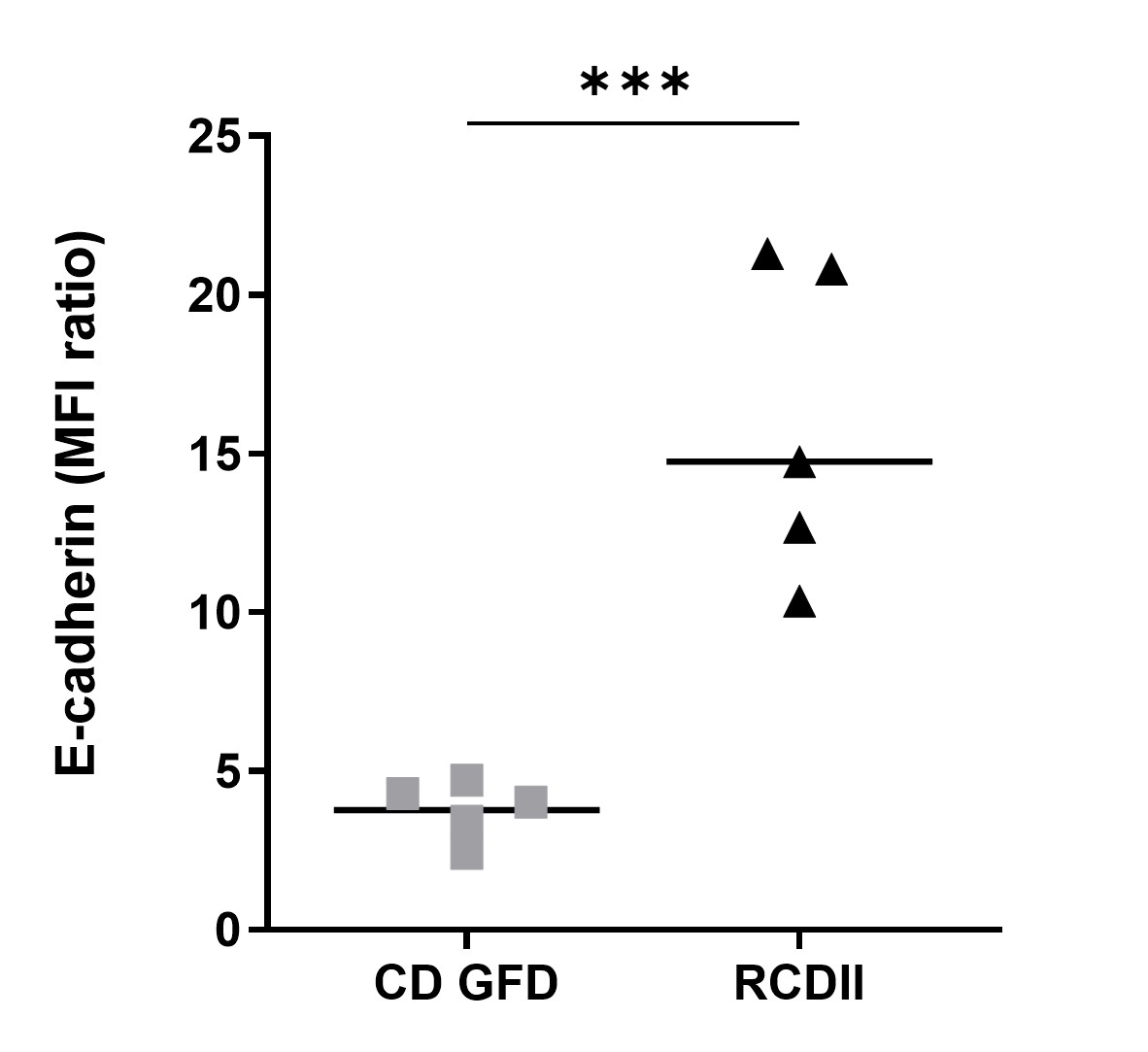

Supplement: Supplementary file 4 — Supplementary figure 4 [file CTI2-15-e70099-s002.jpg]
