# Supplementary material for: Aberrant intra‐epithelial lymphocytes cause enterocyte cell death in refractory celiac disease by CD103‐β7‐receptor‐mediated granzyme‐B degranulation which can be restored by etrolizumab
Source: Clin Transl Immunology. 2026 May 14;15(5):e70099. doi: 10.1002/cti2.70099 (PMC13175919; doi:10.1002/cti2.70099)
Supplement: Supplementary file 5 — Supplementary table 1 [file CTI2-15-e70099-s005.docx]

**Supplemental Table 1. Patient characteristics.**

|  | **Figure 1 A/B** | | **Figure 1C/D** | | | **Figure 3C** | | **Figure 3F/G** | |
| --- | --- | --- | --- | --- | --- | --- | --- | --- | --- |
|  | RCDII,  n=10 | CD on GFD, n=12 | RCDII responder, n=6 | | RCDII non-responder, n=8 | RCDII, n=8 | CD on GFD, n=8 | RCDII, n=8 | CD on GFD, n=10 |
| Male/female, n | 6/4 | 7/5 | 5/1 | | 5/3 | 4/4 | 2/6 | 5/3 | 4/6 |
| Age, years | 67  [51 – 77] | 58 [27 – 75] | 67  [56 – 78] | | 67  [53 – 78] | 70 [63 – 78] | 61 [45 – 71] | 65  [59 – 78] | 50  [18 – 74] |
| Aberrant IEL, % | 53 [20 – 85] | 6.1 [0.2 – 14] | 41  [1 – 46] | | 53 [36 -89] | 53 [34 – 90] | 7 [1 – 19] | 58  [23 – 96] | 8  [3 – 16] |
| Marsh classification, n | | | | | | | |  |  |
| Marsh 0 | 0 | 6 | 6 | | 0 | 2 | 6 | 0 | 10 |
| Marsh 1 | 1 | 6 | 0 | | 0 | 0 | 2 | 0 | 0 |
| Marsh 2 | 3 | 0 | 0 | | 3 | 0 | 0 | 0 | 0 |
| Marsh 3A-C | 6 | 0 | 0 | | 5 | 6 | 0 | 8 | 0 |
| tTG-IgA titer, n | | | | | | | |  |  |
| Negative | 10 | 10 | 6 | | 8 | 8 | 8 | 8 | 10 |
| Weakly positive | 0 | 2 | 0 | | 0 | 0 | 0 | 0 | 0 |
| Positive | 0 | 0 | 0 | | 0 | 0 | 0 | 0 | 0 |
| Prior therapy | | | | | | | | | |
| None | 0 | 0 | 0 | 0 | | 0 | 0 | 0 | 0 |
| GFD | 8 | 12 | 0 | 0 | | 1 | 8 | 7 | 10 |
| Prednisone + azathioprine | 0 | 0 | 1 | 0 | | 0 | 0 | 0 | 0 |
| 2-CDA | 2 | 0 | 3 | 6 | | 7 | 0 | 1 | 0 |
| Autologous SCT | 0 | 0 | 2 | 2 | | 0 | 0 | 0 | 0 |

RCDII = refractory celiac disease type II, CD = celiac disease, GFD = gluten free diet, tTG-IgA = tissue transglutaminase IgA, 2-CDA = 2-chlorodeoxyadenosine / cladribine, SCT = stem cell transplantation.
